# Retinal arterial blood flow and retinal changes in patients with sepsis: preliminary study using fluorescein angiography

**DOI:** 10.1186/s13054-017-1676-3

**Published:** 2017-04-10

**Authors:** Kristo Erikson, Janne Henrik Liisanantti, Nina Hautala, Juha Koskenkari, Remi Kamakura, Karl Heinz Herzig, Hannu Syrjälä, Tero Ilmari Ala-Kokko

**Affiliations:** 1 Division of Intensive Care Medicine, Research Group of Surgery, Anesthesiology and Intensive Care Medicine, Department of Anesthesiology, Oulu University Hospital, Medical Research Center Oulu, University of Oulu, Oulu, Finland; 2 Department of Ophthalmology, Medical Research Center and PEDEGO Research Unit, University of Oulu and Oulu University Hospital, Oulu, Finland; 3 Department of Infection Control, Oulu University Hospital, Medical Research Center Oulu, University of Oulu, Oulu, Finland; 4 Institute of Biomedicine and Biocenter of Oulu, University of Oulu, Oulu, Finland; 5 Medical Research Center Oulu and Oulu University Hospital, Oulu, Finland; 6 Department of Gastroenterology and Metabolism, Poznan University of Medical Sciences, Poznan, Poland

**Keywords:** Sepsis, Retinal arterial blood flow, Retinal changes

## Abstract

**Background:**

Although tissue perfusion is often decreased in patients with sepsis, the relationship between macrohemodynamics and microcirculatory blood flow is poorly understood. We hypothesized that alterations in retinal blood flow visualized by angiography may be related to macrohemodynamics, inflammatory mediators, and retinal microcirculatory changes.

**Methods:**

Retinal fluorescein angiography was performed twice during the first 5 days in the intensive care unit to observe retinal abnormalities in patients with sepsis. Retinal changes were documented by hyperfluorescence angiography; retinal blood flow was measured as retinal arterial filling time (RAFT); and intraocular pressure was determined. In the analyses, we used the RAFT measured from the eye with worse microvascular retinal changes. Blood samples for inflammation and cerebral biomarkers were collected, and macrohemodynamics were monitored. RAFT was categorized as prolonged if it was more than 8.3 seconds.

**Results:**

Of 31 patients, 29 (93%) were in septic shock, 30 (97%) required mechanical ventilation, 22 (71%) developed delirium, and 16 (51.6%) had retinal angiopathies, 75% of which were bilateral. Patients with prolonged RAFT had a lower cardiac index before (2.1 L/kg/m^2^ vs. 3.1 L/kg/m^2^, *P* = 0.042) and during angiography (2.1 L/kg/m^2^ vs. 2.6 L/kg/m^2^, *P* = 0.039). They more frequently had retinal changes (81% vs. 20%, *P* = 0.001) and higher intraocular pressure (18 mmHg vs. 14 mmHg, *P* = 0.031). Patients with prolonged RAFT had lower C-reactive protein (139 mg/L vs. 254 mg/L, *P* = 0.011) and interleukin-6 (39 pg/ml vs. 101 pg/ml, *P* < 0.001) than those with shorter RAFT.

**Conclusions:**

Retinal angiopathic changes were more frequent and cardiac index was lower in patients with prolonged RAFT, whereas patients with shorter filling times had higher levels of inflammatory markers.

## Background

The retina is considered to be one of the most metabolically active tissues in the body [1]; hence, hypoperfusion might lead to a higher probability of microcirculatory and structural retinal changes. Methods such as advanced retinal photographic imaging techniques and computer-assisted image analysis might be used to characterize, measure, and quantify even subtle variations and abnormalities in the retinal microvasculature [2]. Retinal fluorescein angiography has been used to study microvascular changes during and after cardiac bypass [3] and could be a new instrument for noninvasive observation of retinal microcirculatory changes in association with cerebral microemboli [4, 5].

Microcirculatory alterations are common in sepsis. Sublingual and gastric mucosal microcirculatory abnormalities in animal models with septic shock have been well described [6]. Severe microcirculatory sublingual derangements measured by orthogonal polarization spectral or sidestream dark-field imaging techniques are proposed to be better predictors of outcome than global hemodynamic variables [7, 8]. Furthermore, the cerebral microcirculation was impaired in animal models of sepsis [9]. In humans, proinflammatory and anti-inflammatory cytokines are released after microbial invasion with excessive sympathetic outflow. The proinflammatory response is responsible for the hemodynamic consequences of sepsis, namely vasodilation and hyperdynamic state or even myocardial depression [10, 11]. Patients have low resistance, high cardiac output circulation, tachycardia, and hypotension.

In this single-center preliminary study of sepsis in an intensive care unit (ICU) population, we measured retinal blood flow using retinal fluorescein angiography. We hypothesized that alterations in retinal blood flow visualized by angiography may relate to macrohemodynamics, levels of inflammatory mediators, and retinal microvascular pathologies and reflect brain dysfunction or the clinical severity of sepsis.

## Methods

### Setting

This prospective, observational study was performed at a tertiary teaching university hospital (Oulu University Hospital) with a mixed medical-surgical ICU. All patients admitted to the ICU for sepsis or septic shock between 1 January 2012 and 31 December 2014 were screened for participation in this study, which included retinal angiography, measurements of retinal arterial filling time (RAFT), intraocular pressure (IOP), screening for delirium, and measuring blood proinflammatory and cerebral markers as well as hemodynamic parameters. Delirium was assessed using the Confusion Assessment Method for the ICU [12]. Level of consciousness was assessed with the Richmond Agitation-Sedation Scale (scores range from −5 to 4, with lower scores indicating less arousal, higher scores indicating more agitation, and 0 indicating an alert and calm state) [13]. The Glasgow Coma Scale was used to determine the level of consciousness of unsedated patients during imaging.

The inclusion criteria for patients were sepsis and septic shock according to the Surviving Sepsis Guidelines [14]. Patients with acute brain disease, psychiatric disorders, chronic alcoholism, or other types of encephalopathy, as well as those with various ophthalmological conditions, such as age-related macular degeneration, diabetic retinopathy, cataract, angle-closure glaucoma, or eye injuries, were excluded. The study was approved by the local ethics committee of the Northern Ostrobothnia Hospital District. After written informed consent was obtained from the patients or their relatives, patients were included in the study.

### Patient management during the ICU stay

Patients with sepsis and septic shock were managed according to the Surviving Sepsis Campaign guidelines [14] and were administered antibiotic treatment according to our ICU antibiotic stewardship protocol. Macrohemodynamics were monitored by measuring arterial blood pressure and pulmonary arterial pressure. An ICU data management system (Centricity Critical Care*(8.1) SP7 (8.17.034); GE Healthcare, Barrington, IL, USA) was used to collect data concerning daily laboratory results, hemodynamic parameters, need for vasoactive or sedative agents, length of stay (LOS), presence of delirium, and severity-of-illness scoring (Acute Physiology and Chronic Health Evaluation II [APACHE II], Simplified Acute Physiology Score [SOFA]) after inclusion in the study. Surviving patients were met at the follow-up clinic 3–6 months after hospital discharge.

### Interventions

#### Retinal angiography

Standardized retinal fluorescein angiographs and digital images were obtained for both eyes. Mydriasis was achieved 30 minutes after instilling topical tropicamide and phenylephrine hydrochloride eye drops. A Heidelberg Retina Angiograph 2 (HRA 2) camera was used in exceptional vertical alignment (HRA 2-00153; Heidelberg Engineering, Heidelberg, Germany). After 4 minutes, postangiography images were taken. The phase identified by laminar flow in the veins was determined as the endpoint of the arterial filling stage and start of the venous phase. The arterial filling time was measured from the patient’s right eye. The preliminary images were taken on the first possible day of the ICU stay or if the inclusion criteria were fulfilled during the ICU stay. The patients were reexamined 2–5 days later. The third angiography was performed 3–6 months after the hospital discharge. These postdischarge images were used as within-patient controls to detect pathological findings in fundus images during the ICU stay. The images were analyzed by an ophthalmologist blinded to the patients’ identity and any clinical data to interpret pathological findings.

#### Arterial filling time

RAFT was determined on the basis of digital fluorescein angiography using the HRA 2. In the analyses, we used the RAFT measured from the eye with worse microvascular retinal changes. The initial phase of the fluorescein angiography was determined, and the early filling stages were documented. The arterial phase was started when the central retinal artery began to fill. The median arterial filling time of the study population was 8.3 seconds. This median value was used as a cutoff point to divide the patient population into two groups: short retinal arterial filling time (SRAFT) less than 8.3 seconds and prolonged retinal arterial filling time (PRAFT) greater than 8.3 seconds.

#### Intraocular pressure measurement

IOP was measured in both eyes using an Icare PRO tonometer (1201775 TA 03 Icare® PRO; Icare Finland Oy, Vantaa, Finland). IOP measurements were performed at the time of each angiography and after 3–6 months at the post-ICU clinic. In patients with retinal abnormalities, the IOP in the eye with the most intense findings was included in the analyses, whereas the first measurement from the right eye was used for those patients without retinal abnormalities.

### Laboratory data

Separated plasma was stored at −70 °C. The inflammatory cytokines tumor necrosis factor-α (TNF-α) and interleukin 6 (IL-6) as well as markers of brain dysfunction (neuron-specific enolase [NSE] and calcium-binding protein B [S100B]) were analyzed. Cytokine concentrations were determined using the MILLIPLEX® MAP Human Cytokine/Chemokine Magnetic Bead Panel (HCYTOMAG-60 K; EMD Millipore, Billerica, MA, USA). The lower detection limits were 0.7 pg/ml for TNF-α and 0.9 pg/ml for IL-6. The intra-assay coefficients of variation (CVs) for TNF-α and IL-6 were 2.6% and 2.0%, respectively, and the corresponding inter-assay CVs were 13% and 18.3%, respectively. S100B and NSE were measured using an immunochemiluminometric method (Elecsys 2010 analyzer; Roche Diagnostics GmbH, Mannheim, Germany).

### Statistical analysis

The statistical analysis was performed using IBM SPSS Statistics version 22 software (IBM, Armonk, NY, USA). Proportional data are expressed as rate (count) and percent, and continuous variables are expressed as median and 25th and 75th percentiles. Proportional data were tested using Pearson’s chi-square test unless otherwise stated. Continuous variables were tested using a nonparametric Mann-Whitney *U* test and independent samples median test. Two-tailed *P* values less than 0.05 were considered statistically significant.

### Outcome measures

The primary outcome measure was median RAFT. The secondary outcome endpoints were the IOP measurements and their relationship to RAFT and different pathological findings in the retina during sepsis. Also, demographic data, comorbidities, severity scores, and outcome data were compared between the SRAFT and PRAFT patient groups.

## Results

During the 2012–2015 study period, a total of 667 patients with sepsis were screened for the present study. Of those, 342 patients met the study criteria, and 309 were admitted outside office hours or during times when there was no possibility for retinal angiography to be performed, leaving 33 patients who were included in the study. Two patients later refused to participate, leaving 31 patients for the final analysis.

The majority of the study population was male (20 males, 11 females). The median age was 62.1 (50.6–75.8) years. The median APACHE II score was 22 (18–25). Retinal abnormalities were observed in 16 patients (51.6%), 12 (75.0%) of whom had bilateral changes. The 30-day mortality was 12.9% (4 of 31). The median RAFT was 8.3 (6.1–10.8) seconds (Table 1).

**Table 1:** Demographic and clinical characteristics of the study population

	All included patients (*N* = 31)	Patients with arterial filling time <8.28 seconds (*n* = 15)	Patients with prolonged arterial filling time >8.28 seconds (*n* = 16)	*P* value
Age, years	62.1 [50.6–75.8]	62.1 [50.6–78.0]	62.3 [52.3–73.8]	0.65
Sex, male/female	20/11	11/5	9/6	0.61
BMI, kg/m^2^	30.5 [25–36]	29.5 [23.6–33.6]	31.1 [26.1–35.9]	0.29
APACHE II score	22 [18–25]	20 [15–21]	22.5 [21.2–29.5]	0.049
APACHE score >25, *n* (%)	6 (19.4)	2 (13.3)	4 (25)	0.41
SAPS II	48 [43–59]	46 [42–59]	53 [46.2–61.7]	0.22
SOFA score at admission	8 [5–10]	7 [5–10]	8 [6.2–9.7]	>0.9
SOFA score, maximum	10 [8–14]	10 [8–12]	12 [8.2–14.7]	0.12
Coronary artery disease, *n* (%)	7 (22.6)	2 (13.3)	5 (31.3)	0.23
Arteriosclerosis obliterans, *n* (%)	4 (12.9)	2 (13.3)	2 (12.5)	0.94
Diabetes dietary, *n* (%)	10 (32.3)	6 (40)	4 (25)	0.37
Chronic kidney disease, *n* (%)	2 (6.5)	2 (13.3)	0	0.13
Chronic obstructive pulmonary disease, *n* (%)	5 (16.1)	3 (20)	2 (12.5)	0.57
Operated, *n* (%)	6 (19.4)	5 (33.3)	1 (6.3)	0.172^a^
Mechanical ventilation, *n* (%)	30 (96.8)	14 (93.3)	16 (100)	0.48
ARRT, *n* (%)	11 (35.5)	5 (33.3)	6 (37.5)	0.81
Use of sepsis corticosteroid, *n* (%)	20 (64.5%)	10 (66.7)	10 (62.5)	0.81
Positive blood cultures, *n* (%)	10 (33.3)	6 (40)	4 (26.7)	0.44
Pneumonia, *n* (%)	9 (29)	4 (26.7)	5 (31.3)	0.78
Abdominal, *n* (%)	12 (38.7)	6 (40)	6 (37.5)	0.89
Genitourinary, *n* (%)	3 (9.7)	1 (6.7)	2 (12.5)	0.58
Soft tissue, *n* (%)	4 (12.9)	1 (6.7)	3 (18.8)	0.31
Unknown, *n* (%)	4 (12.9)	2 (13.3)	2 (12.5)	0.94
Endocarditis, *n* (%)	1 (3.23)	1 (6.25)	0	0.29
Delirium, *n* (%)	22 (71)	10 (66.7)	12 (75)	0.77
Cumulative fluid balance during angiography, ml	421 [−1502 to 3343]	1409 [−841 to 46 06]	−620 [−1533 to 3015]	0.44
ICU LOS, survivors, days	7.6 [3.3–14]	5.9 [3.3–9.6]	7.8 [3.3–9.4]	0.72
Hospital LOS, days	20 [8.5–35]	22.9 [10.8–34.7]	19.3 [7.9–32.3]	0.49
ICU mortality, *n* (%)	3 (9.7)	2 (12.5)	1 (6.7)	>0.9
30-day mortality, *n* (%)	4 (12.9)	1 (6.7)	3 (18.8)	0.32^a^
365-day mortality, *n* (%)	8 (25.8)	3 (20)	5 (31.3)	0.38^a^

*Abbreviations: APACHE II* Acute Physiology and Chronic Health Evaluation II, *ARRT*, Acute renal replacement therapy, *ICU*, Intensive care unit, *LOS*, Length of stay, *SAPS II*, Simplified Acute Physiology Score II, *SOFA*, Sequential Organ Failure Assessment, *BMI*, Body mass index

^a^ *P* value calculated using Fisher’s exact test

Patients with arterial PRAFT had higher APACHE II scores (22.5 [21.2–29.5] vs. 20 [15–21], *P* = 0.049), and their IOP was higher (17.8 [14.7–22.1] mmHg vs. 14 [12.5–15.5] mmHg, *P* = 0.029). Retinal abnormalities were more frequent in those with PRAFT (81% vs. 20%, *P* = 0.001) (Table 1).

**Fig. 1:**
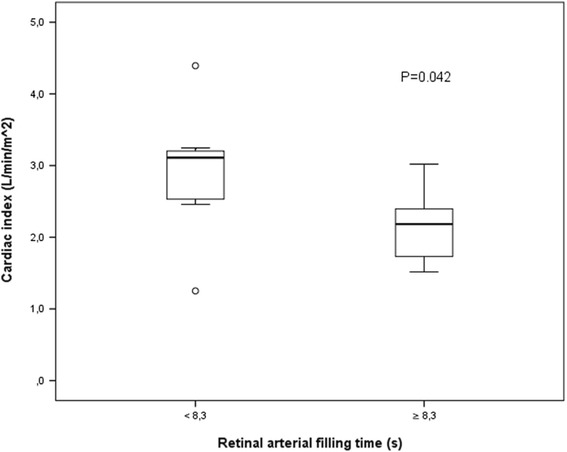
Cardiac index according to retinal arterial filling time measured by fluorescein angiography

Cardiac index measured with a thermodilution technique using a pulmonary catheter was lower in those with PRAFT before angiography (2.1 [1.7–2.5] vs. 3.1 [2.5–3.2], *P* = 0.042) and at angiography (2.1 [1.7–2.4] vs. 2.6 [2.2–3.1], *P* = 0.039). There were no differences in the doses of vasopressors, mean arterial pressures, or serum lactate levels between the PRAFT and SRAFT groups during angiography (Table 2, Fig. 1).

**Table 2:** Retinal fluorescein angiography: retinal changes, ocular pressure, tissue perfusion parameters, and inflammatory markers

	All patients (*N* = 31)	Patients with short arterial filling time <8.28 seconds (*n* = 15)	Patients with prolonged arterial filling time >8.28 seconds (*n* = 16)	*P* value
Intraocular pressure at angiography, mmHg	15.7 [13.3–19.5]	14.0 [12.5–15.5]	17.8 [14.7–22.1]	0.031
Retinal changes, *n* (%)	16 (51.6)	3 (20)	13 (81.3)	0.001
Mean blood glucose level, mmol/L	7.8 [6.8–10.4]	8.9 [7.7–10.2]	8.1 [7.2–9.4]	0.36
CRP at angiography, mg/L	160 [114–316]	254 [146–366]	139 [77.7–224.5]	0.011
CRP, highest before angiography, mg/L	264 [149–380]	307 [161.7–435.7]	187 [99.5–363.5]	0.104
PCT at angiography, μg/L	3.87 [1.96–12.2]	0.387 [1.96–12.2]	7.06 [1.9–31.8]	0.28
PCT, maximum before angiography, μg/L	9.4 [2.4–36.6]	11.6 [6.7–30.3]	6.7 [0.96–61.9]	0.669
Platelets, lowest before angiography, ×10^9^/L	170 [124–219]	184.5 [166–270.7]	146 [98.5–202.3]	0.056
Platelets, lowest at angiography, ×10^9^/L	149 [90–185]	167 [123–209]	96 [84–178]	0.07
PT-INR, highest before angiography	1.3 [1.15–1.9]	1.2 [1.1–1.6]	1.45 [1.27–2.2]	0.025
PT-INR, highest at angiography	1.35 [1.2–1.67]	1.25 [1.17–1.45]	1.45 [1.2–2.1]	0.09
Noradrenaline dose, highest before angiography, μg/kg/minute	0.2 [0.06–0.53]	0.23 [0.073–0.64]	0.167 [0–0.38]	0.47
Noradrenaline dose during angiography, μg/kg/minute	0.04 [0–0.24]	0.0242 [0–0.61]	0.050 [0.005–0.23]	0.71
Cardiac index, lowest before angiography, L/minute/m^2^	2.4 [1.7–3]	3.1 [2.5–3.2]	2.1 [1.7–2.5]	0.042
Cardiac index, during angiography, L/minute/m^2^	2.3 [1.9–2.7]	2.6 [2.2–3.1]	2.1 [1.7–2.4]	0.039
MAP at angiography, mmHg	73.9 [68–88]	78.5 [69.5–88.8]	69.6 [65.5–89.2]	0.49
MAP below 65 mmHg before angiography, minutes	13.6 [8–33]	11 [4.5–37.5]	23 [8–33]	0.470
Lactate angiography, mmol/L	1.9 [1.3–2.5]	1.75 [1.31–2.5]	1.98 [1.34–2.54]	0.62
Lactate, highest before angiography, mmol/L	3.4 [1.9–8.6]	2.97 [2.2–6.7]	6.05 [1.87–10.03]	0.61
BE lowest before angiography, mmol/L	−7.8 [−13.5 to −4.8]	−7.8 [−12.9 to −3.3]	−7.9 [−14.1 to −7]	0.467
BE during angiography, mmol/L	−4.3 [−7.9 to 0.375]	−3.2 [−7.5 to 0]	−4.9 [−11.1 to 0.9]	0.57
PaO_2_, mmHg	12.3 [11.4–14.5]	11.4 [10.3–13.8]	12.2 [9.9–13]	0.66
PaCO_2_, mmHg	4.9 [4.5–6]	5.2 [4.6–6]	5.1 [4.2–5.9]	0.77
PaO_2_/FiO_2_ ratio, mmHg	31.5 [23.6–37.1]	34.6 [23.8–47.9]	34.2 [23.8–43.7]	0.95

*Abbreviations: CRP*, C-reactive protein, *PCT*, Procalcitonin, *PT-INR*, Prothrombin time international normalized ratio, *MAP*, Mean arterial pressure, *BE*, Base excess, *PaCO*_*2*_, Partial pressure of carbon dioxide in arterial blood, *PaO*_*2*_ */FiO*_*2*_ *ratio* Ratio of partial pressure of oxygen in arterial blood to fraction of inspired oxygen

Patients with PRAFT had a lower C-reactive protein (CRP) level on the day of angiography (139 [77.7–224.5] mg/L vs. 254 [146–366] mg/L, *P* = 0.011) (Table 2). Patients with PRAFT had significantly lower levels of NSE, S100B, TNF-α, and IL-6 than patients with SRAFT (Table 3).

**Table 3:** Inflammatory and cerebral markers at angiography and at the intensive care unit follow-up clinic

	Patients with short arterial filling time <8.28 seconds (*n* = 15)	Patients with prolonged arterial filling time >8.28 seconds (*n* = 16)	ICU follow-up clinic	*P* value
S100B, μg/L	0.19 [0.097–0.45]	0.12 [0.06–0.22]	0.04 [0–0.05]	<0.001
NSE, μg/L	23.5 [13.3–39.2]	22.3 [8.8–34.2]	13.4 [0.82–17.2]	0.035
IL-6, pg/ml	101 [13.4–525.6]	39.4 [19.6–77.6]	3.7 [0.58–8.56]	<0.001
TNF-α, pg/ml	29.5 [20.2–82.5]	28.3 [19.5–49]	13.9 [9.3–20.8]	0.002

*Abbreviations: ICU* Intensive care unit, *IL-6* Interleukin 6, *NSE* Neuron-specific enolase, *S100B* Calcium-binding protein B, *TNF-α* Tumor necrosis factor-α

**Fig. 2:**
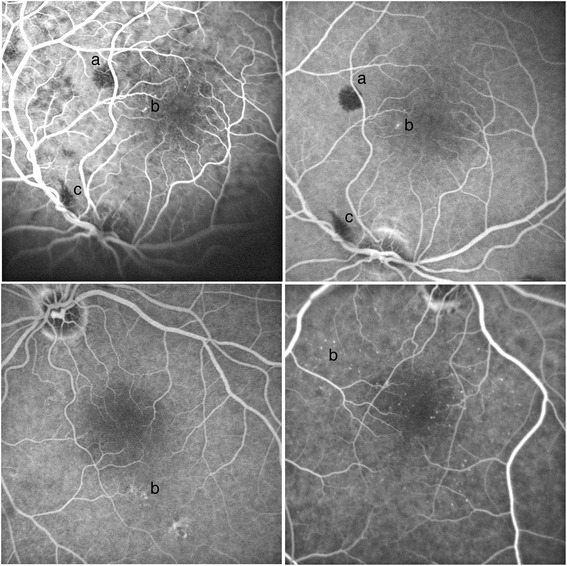
Retinal changes in patients with sepsis. (retinal hemorrhages (**a**), micoaneurysms (**b**) and vitreous hemorrhages (**c**))

Pathological retinal findings were present in 16 patients (51.2%) (Fig. 2). The most common retinal abnormality was fluorescein-leaking retinal microaneurysm (56%), followed by vitreous hemorrhage (13%) and other retinal hemorrhages (6.5%). The abnormal retinal findings were present in 13 (81.3%) of 16 patients in the PRAFT group and 3 (20.0%) of 15 patients in the SRAFT group (*P* = 0.001). In surviving patients, the abnormal retinal findings were found to have resolved in control angiography.

The IOP was higher in the prolonged RAFT group (18 mmHg vs. 14 mmHg, *P* = 0.031). Interestingly, 16% of all the patients with sepsis studied had ocular hypertensive pressures (IOP >21 mmHg). There were no differences in ICU, 30-day, or 365-day mortality rates between the two patient groups with different arterial filling times (Table 1). In the control angiography of the survivors performed 3–6 months following ICU discharge, the median RAFT was 12.56 (12.1–14.9) seconds, and there were no differences between the PRAFT and SRAFT groups.

## Discussion

In the present preliminary prospective study, we found that, first, patients with sepsis and PRAFT had retinal abnormalities and experienced higher IOPs more frequently than those with sepsis and SRAFT. Second, patients in the PRAFT group had a significantly lower cardiac index and lower IL-6 and CRP levels. Third, these patients had higher APACHE II scores upon admission.

To the best of our knowledge, this is the first study involving measurement of retinal blood flow in human patients with sepsis and exploration of the feasibility of this measurement to reflect the macro- and microcirculation. The decision to use median time as the cutpoint value for prolonged RAFT could be considered a limitation of the present study. Because there are no reference values for RAFT, and owing to the limited sample size, we decided to use the median in this pilot study. Patients with SRAFT had higher cardiac indexes and more elevated levels of inflammatory markers. This is in line with the hyperdynamic hemodynamic state often seen in early sepsis with a proinflammatory phase [15]. In contrast, the relationship between the cardiac index and microcirculatory perfusion has not been demonstrated using sublingual techniques [16, 17]. However, in another study with an early goal-directed fluid therapy and vasoactive medication protocol, sublingual microcirculation flow velocity correlated with mean arterial pressure and mixed venous oxygen saturation [18]. According to an animal model of hyperdynamic septic shock, arterial flow is increased in the mesenterial artery, whereas ileal microcirculation is decreased. Taken together, human organ blood flow distribution varies between different organs in patients with sepsis, and according to our results, it follows changes in cardiac output.

Our results suggest that retinal angiography may be used to estimate the macrocirculation and could provide a new technique for noninvasive monitoring of the central nervous system. These findings generate ideas for future studies on the applicability of retinal blood flow as a tool for the assessment of microcirculation and cerebral circulation in particular. In our series, prolonged retinal blood flow was related to retinal microcirculatory abnormalities. Further studies are needed to evaluate whether retinal blood flow changes are related to systemic microhemodynamics. It may be possible to evaluate the severity of illness by simply looking into the patient’s eye with a fundus camera, which is accessible and also easily affordable for the ICU clinician.

In the present series, the IOP was higher among patients with PRAFT. Similarly, correlation between increased IOP and delayed filling time of the retinal veins was suggested in an earlier study in patients with glaucoma [19, 20]. Decreased IOP was previously reported in patients with Puumala virus infections, with a mean IOP of 4.5 mmHg [21]. To our knowledge, intraocular hypertension has not previously been documented in patients with sepsis. In our series, 16% had IOP >21 mmHg.

In our study, retinal abnormalities were observed in half of the patients with sepsis and were more common in patients in the PRAFT group (81%). Microvascular changes in the retina are usually associated with diabetes and hypertension [22]. In our series of patients admitted to the ICU with sepsis, we found fluorescein-leaking retinal microaneurysms and retinal hemorrhages. In harmony with our results, intra- and periretinal hemorrhages were observed in a previous study of patients with severe acute pancreatitis [23]. In that study, retinal changes were associated with multiple organ failure syndrome. In our series, patients with retinal changes were more severely ill on admission (APACHE II score 22.5 [21.2–29.5] vs. 20 [15–21], *P* = 0.049).

A novel finding of the present study is the fluorescein-leaking retinal microaneurysms. Pathological processes such as inflammation or ischemia may upset the normal retina-blood barrier in the retinal capillaries, thereby allowing extravascular leakage of fluorescein. Even transient hypoxia, for example, can increase the permeability of the retinal arterioles even with normal intraluminal blood pressure and may also later involve the venous side [24]. These permeability changes are thought to be related to changes in the vascular endothelial cells. In our patients, sepsis-related lower cardiac indexes and elevated IOPs with altered, prolonged retinal blood flow may have produced transient retinal hypoxia and may have resulted in the development of retinal microvascular abnormalities. In our study, inflammatory parameters measured (IL-6, CRP) at the same time as retinal blood flow were significantly higher in those with shortened retinal filling time. In our series, the rapid retinal blood flow could thus be linked with the hyperdynamic hemodynamic and proinflammatory responses.

### Limitations

The main limitation of the present study is the relatively small number of patients. This was due to the study design, with highly complex interventions and measurements performed only during office hours. The sample size was insufficient to detect clinically relevant patient-centered outcomes. The results are observational, descriptive, and hypothesis-generating in nature. Second, our cutoff point of 8.3 seconds was the median filling time. The literature concerning filling time is sparse. Arterial filling time in the retina has not been used previously to measure blood flow in patients with sepsis. However, we were able to show differences between patients with prolonged and shorter filling times, even in this relatively small patient population. In future studies in this field, researchers should determine the reference values for RAFT in patients with sepsis. Further studies are also needed to determine whether retinal angiographic changes are related to, for example, sublingual microcirculatory alterations. It is notable that the mortality in the present series was relatively low when considering that almost all the patients had septic shock.

Another possible limitation of the study is that the imaging was performed in different phases of the ICU stay in different patients. This was due to complex interventions and measurements performed only during office hours. However, it is also true that it is not possible to harmonize the timing of the intervention in this patient material, because patients are also admitted to the ICU in different phases of the disease, depending on the severity of the disease, the type of infection, and the symptoms.

## Conclusions

This preliminary study demonstrates that retinal angiography could be a feasible monitoring technique for the ICU. The retinal blood flow, IOP, and microvascular angiopathies were linked in the patients with sepsis. Patients with RAFTs more than 8.3 seconds had a lower cardiac index and more frequently had retinal angiopathies, higher IOP, and lower levels of inflammatory markers, indicating an impaired inflammatory response.

## Abbreviations

APACHE II: Acute Physiology and Chronic Health Evaluation II
ARRT: Acute renal replacement therapy
BE: Base excess
BMI: Body mass index
CRP: C-reactive protein
CV: Coefficient of variation
FiO_2_: Fraction of inspired oxygen
HRA 2: Heidelberg Retina Angiograph 2
ICU: Intensive care unit
IL-6: Interleukin 6
IOP: Intraocular pressure
LOS: Length of stay
MAP: Mean arterial pressure
NSE: Neuron-specific enolase
PaCO_2_: Partial pressure of carbon dioxide in arterial blood
PaO_2_: Partial pressure of oxygen in arterial blood
PaO_2_/FiO_2_: Ratio of partial pressure of arterial oxygen to fraction of inspired oxygen
PCT: Procalcitonin
PRAFT: Prolonged retinal arterial filling time
PT-INR: Prothrombin time international normalized ratio
RAFT: Retinal arterial filling time
S100B: Calcium-binding protein B
SAPS II: Simplified Acute Physiology Score II
SOFA: Sequential Organ Failure Assessment
SRAFT: Short retinal arterial filling time
TNF-α: Tumor necrosis factor-α
